# Radical resection and enucleation in Chinese adolescents with pancreatic tumors: A 15-year case series

**DOI:** 10.1097/MD.0000000000006438

**Published:** 2017-03-24

**Authors:** Lie Yao, Zhi-Bo Xie, Chen Jin, Yong-Jian Jiang, Ji Li, Feng Yang, Quan-Jun Lin, De-Liang Fu

**Affiliations:** Department of Pancreatic Surgery, Pancreatic Disease Institute, Huashan Hospital, Shanghai Medical College, Fudan University, Shanghai, China.

**Keywords:** adolescent, growth, pancreatic tumor, radical resection, survival

## Abstract

Pancreatic tumors rarely occur in adolescents, and the appropriateness of radical resection for these patients remains controversial.

Medical records were retrospectively reviewed for patients younger than 19 years who underwent radical resection or limited resection (enucleation) between 2000 and 2015. Patient demographics, clinical characteristics, operative details, growth, and survival were analyzed.

During the study period, 11 adolescents (mean age, 16.18 years; standard deviation, 1.99; interquartile range, 15.0–18.0) underwent radical resection (n = 7) or enucleation (n = 4) to treat solid pseudopapillary tumors (n = 5), pancreatic neuroendocrine tumors (n = 5), or pancreatic ductal adenocarcinoma (n = 1). None of the 7 patients who underwent radical resection experienced recurrence or serious complications, while 3 of 4 patients who underwent enucleation experienced recurrence (*P* = 0.02). Recurrence-free survival was slightly longer in patients who underwent radical resection, and this procedure did not appear to affect adolescent growth and development.

Radical resection might be safe and effective for adolescents with pancreatic tumors.

## 1 Introduction

Pancreatic tumors rarely occur in adolescents,^[1–3]^ and overall survival (OS) of adolescent patients is better than that of adults.^[4]^ Pancreatic tumors reported in pediatric patients include pancreatoblastomas, acinar cell carcinomas, pancreatic ductal adenocarcinomas (PDACs), solid pseudopapillary tumors (SPTs), pancreatic neuroendocrine tumors (PNETs), sarcomas, lymphomas, undifferentiated carcinomas, and cystadenocarcinomas.^[5–7]^

Radical resection is a standard curative therapy routinely applied to adults with pancreatic tumors, in whom it can significantly extend OS.^[8]^ While some studies indicate that radical resection can also be effective in adolescents, leading to negligible risk of recurrence.^[9,10]^ However, some clinicians may be reluctant to use it for fear of complications or poor prognosis, especially in young children. In addition, whether radical resection perturbs adolescents’ growth and development remains unclear.

Studies of safety and efficacy,^[11–13]^ each involving 10 to 20 patients, suggest that pediatric pancreatic tumors are rare entities and long-term outcomes are generally good. Even with the presence of positive margins, SPT patients can still show excellent outcomes, suggesting that limited surgical resection may be appropriate.^[13]^ All these studies have been conducted in the West, highlighting the need to examine these questions in Asian healthcare contexts. Therefore we evaluated efficacy and safety outcomes among adolescents treated for pancreatic tumors at our hospital in China over a 15-year period. We also evaluated the long-term influence of radical resection on growth and development.

## 2 Patients and methods

### 2.1 Ethics statement

The study was approved by ethical committees of Huashan Hospital, and it was conducted in accordance with the Declaration of Helsinki and internationally accepted ethical guidelines. The parents or legal guardians of enrolled adolescents signed written consent for their information to be stored in hospital databases and used for research. Records of study participants were anonymized.

### 2.2 Patients and outcomes

Our hospital database was searched for patients younger than 19 years who were treated for pancreatic tumors between 2000 and 2015. All records for those patients were reviewed, including baseline characteristics and perioperative outcomes. Postoperative data on postoperative growth and development were also examined during follow-up examinations every 6 months. Growth and development were assessed against standard height and weight curves in the case of patients younger than 7 years, or against body mass index (BMI) in the case of children between 7 and 19 years old. In the latter case, patients with BMI ≤ 19.9 were defined as underweight; those with BMI of 20 to 24.9, as normal; those with BMI of 25 to 27.9, as overweight; and those with BMI ≥ 28, as obese.^[14]^

Recurrence-free survival (RFS) was defined as the interval from initial treatment until recurrence. If patients had not experienced recurrence by the end of the study period, RFS was considered to be equal to OS.

### 2.3 Statistical analysis

Statistical analysis was performed using SPSS 21.0 (IBM, Chicago, IL), with *P* < 0.05 defined as the threshold of statistical significance. Normally distributed data were expressed as mean ± standard deviation (SD), while asymmetrically distributed data were expressed as median (interquartile range). Intergroup differences in categorical variables were assessed for significance using Fisher exact test or the χ^2^ test.

## 3 Results

### 3.1 Characteristics of the study population

During the 15-year study period, 11 adolescents (mean age, 16.18 years; SD, 1.99; interquartile range, 15.0–18.0 years) were treated at our hospital for SPT (n = 5), PNET (n = 5), or PDAC (n = 1). Median tumor diameter was 2.0 cm (interquartile range, 1.8–7.0). Seven adolescents were initially treated with radical resection and 4 with enucleation. The mean operating time over all 11 patients was 300 ± 121 min, and median blood loss was 300 mL (interquartile range: 200–500). The mean length of hospital stay was 23.2 ± 7.9 days. Recurrence occurred during follow-up in 3 of the 11 patients, all of whom had undergone enucleation (Tables 1 and 2).

**Table 1:**
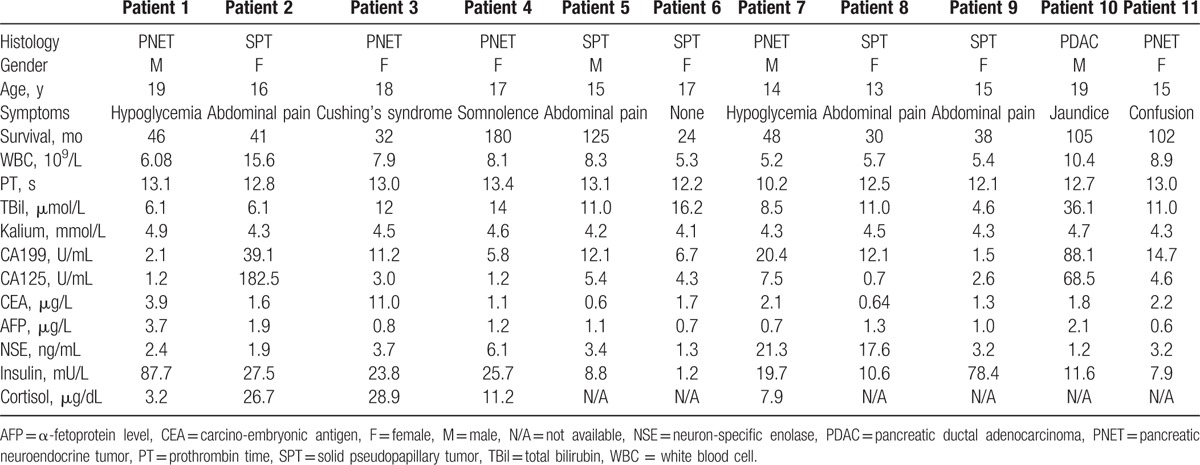
Baseline characteristics of included patients.

**Table 2:**
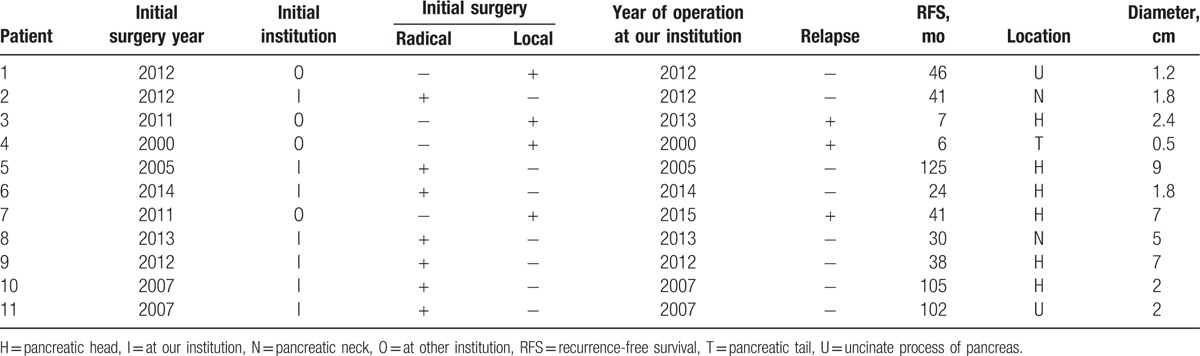
Operative details for included patients.

### 3.2 Analysis of patients with different types of pancreatic tumors

#### 3.2.1 Solid pseudopapillary tumors

Patients 2, 5, 6, 8, and 9 were diagnosed with SPT. The mean age was 15.2 ± 1.5 years. The median OS was 38 months (interquartile range, 27–83), and the median RFS was 38 months (interquartile range, 27–83). The mean operating time was 305.0 ± 107.5 min, median blood loss was 400 mL (range, 175–500) and the mean length of hospital stay was 20.2 ± 7.7 days. None of the patients showed lymph node or vascular invasion, and none experienced recurrence during follow-up.

Patient 2 with SPT had undergone pancreaticoduodenectomy (PD) with external drainage. Postoperative grade A pancreatic fistula occurred, and the patient recovered after 10 days in hospital. Patient 8 underwent central pancreatectomy, and no complications or recurrence occurred during follow-up.

Patients 5, 6, and 9 underwent pylorus-preserving pancreaticoduodenectomy (PPPD). Patient 5 suffered grade A pancreatic fistula, while patient 6 showed fungal enteritis and delayed gastric emptying. No complications were detected in patient 9. All patients recovered after appropriate active therapy. Patient 5 was hospitalized for a total of 20 days; patient 6, 31 days; and patient 9, 17 days.

#### 3.2.2 Pancreatic neuroendocrine tumors

Patients 1, 3, 4, 7, and 11 were diagnosed with PNET. Patient 3 was diagnosed at another medical center in 2011 with pancreatic adrenocorticotropic hormone adenoma presenting with Cushing syndrome. The patient underwent enucleation and recovered uneventfully. In 2013, the patient, who had become overweight, presented with Cushing syndrome again. He was admitted to our hospital and underwent examinations, which revealed recurrence. The patient underwent classic Whipple surgery, during which the portal vein was found to be invaded. The portal vein was resected and reconstructed, and the patient was diagnosed with pancreatic neuroendocrine carcinoma based on postoperative pathology analysis.

Among patients 1, 4, 7, and 11, the mean age was 16.0 ± 2.2 years, the median OS was 75.0 months (interquartile range, 46.5–160.5) and the median RFS was 44.0 months (interquartile range, 14.8–88.0). These 4 patients had functioning PNETs and suffered hypoglycemia; the median operating time was 183 min (interquartile range, 158–346), median blood loss was 250 mL (range, 88–300) and the mean hospital stay was 23.8 ± 7.5 days. Recurrence occurred in patients 4 and 7 during follow-up.

In 2008, patient 1 suffered hypophysoma with symptoms of high blood pressure; the following year, the hypophysoma was removed from the patient's sella region at another medical center. In 2011, hypoglycemia appeared, and analysis revealed a tumor in the uncinate process of the pancreas. Serum insulin levels were abnormally high and local resection was performed in 2012. After resection, insulin levels returned to normal. The patient subsequently suffered *Acinetobacter baumannii* infection and recovered after a 14-day hospital stay involving antibiotic and other systematic and supportive therapies. The patient did not experience recurrence by the end of the study.

Patient 4 was diagnosed with pancreatic islet cell tumor and underwent local enucleation in January 2002 at another medical center. Postoperative grade B pancreatic fistula occurred. After 6 months, the patient showed abnormal insulin levels again, and analysis revealed PNET recurrence. The patient underwent recurrence enucleation 1 week later. Insulin levels returned to normal after surgery, and no serious complications were observed. The patient was discharged after a 28-day hospital stay and did not experience recurrence by the end of follow-up.

**Figure 1:**
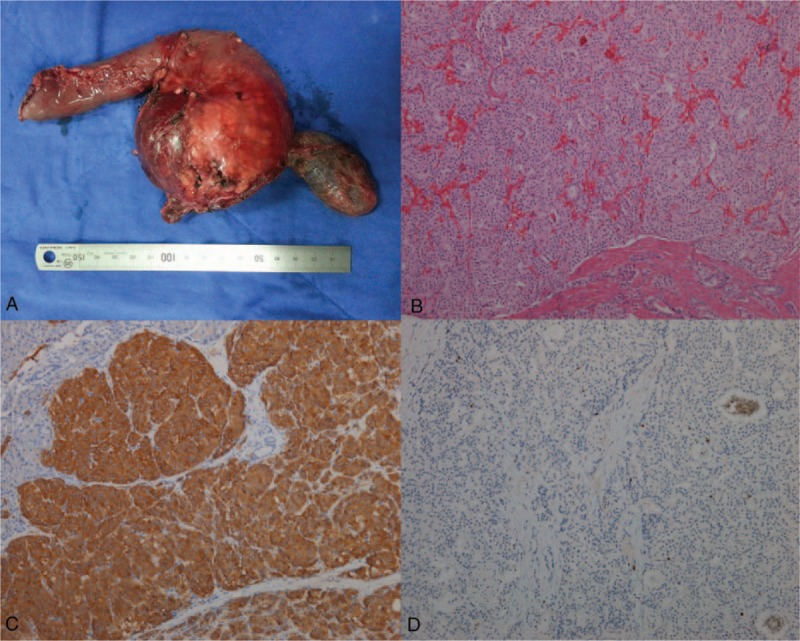
Pathological findings for patient 7. (A) Gross appearance of the tumor. (B) Microscopic appearance of the tumor, after hematoxylin and eosin staining (magnification ×100). (C) Immunohistochemistry revealed positive staining for synaptophysin (magnification ×400). (D) Immunohistochemistry revealed positive staining for Ki67 (magnification ×100).

**Figure 2:**
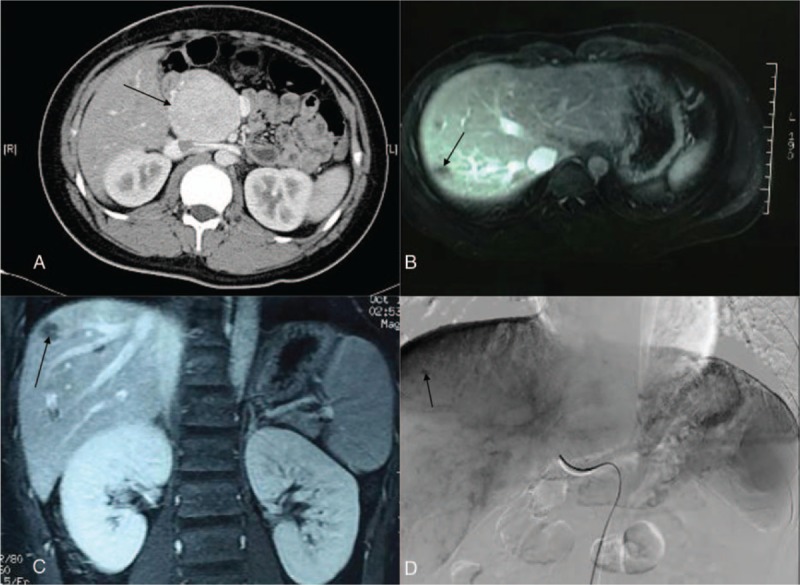
Preoperative images and liver metastasis images of patient 7. (A) Preoperative computed tomography scan showing a 7-cm lesion at the head of the pancreas. (B) Magnetic resonance imaging showing postoperative liver metastasis. (C) Magnetic resonance imaging of the coronary area showing postoperative liver metastasis. (D) Digital subtraction angiograph showing postoperative liver metastasis.

Patient 7 underwent enucleation initially in 2011, followed by PPPD for recurrence in May 2015. No serious complications occurred. Liver metastases were detected 5 months later (Figs. 1 and 2), which were treated using chemotherapy infusion (TAI) of gemcitabine (1.4 g), oxaliplatin (150 mg), and fluorouracil (1.0 g) into the transhepatic artery. Transhepatic artery chemotherapy infusion (TAI) was repeated every 4 weeks, together with somatostatin analog therapy (octreotide acetate microspheres for injection, 20 mg).

Patient 11 underwent resection in 2007. No serious complications occurred, and no recurrence was observed through the end of follow-up.

#### 3.2.3 Pancreatic ductal adenocarcinoma

Patient 10 had symptoms of jaundice and abdominal pain and was diagnosed with PDAC. A classic Whipple procedure was performed, and no obvious complications occurred. Starting on postoperative week 6, TAI was performed using the mixture of gemcitabine, oxaliplatin, and fluorouracil described above. TAI was repeated every 4 weeks for 6 months. The patient did not experience recurrence by the end of follow-up.

### 3.3 Differences between patients undergoing radical or local resection

No relapse was observed among the 7 patients who underwent radical resection, whereas it occurred in 3 of the 4 patients who underwent enucleation (*P* = 0.02). The median RFS in patients underwent radical resection and enucleation is 41 months (range, 30–105) and 24 months (range, 6–45), respectively. None of the 11 patients experienced surgery-induced diabetes mellitus.

### 3.4 Growth and development

Growth and development of the patients were analyzed immediately before surgery and at the end of follow-up (Table 3). At the time of initial surgery, 7 patients showed normal growth (patients 2, 4, 6, 7, 9, 10, and 11); 2 were overweight (patients 1 and 3); and 2 were underweight (patients 5 and 8). In December 2015, all patients showed normal growth except patient 3, who was overweight, and patient 8, who was underweight. Moreover, serum levels of insulin and glucagon were normal. Patient 5 recently married and had a baby, and patient 6 recently married.

**Table 3:**
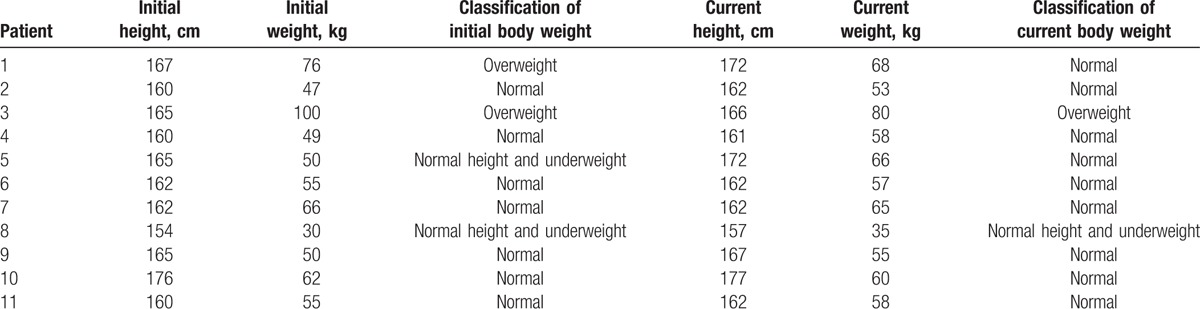
Current growth status of each adolescent (through December 2015).

## 4 Discussion

Among patients aged 0 to 19 years, annual incidence of pancreatic tumors is 0.018 per 100,000 according to the North American population-based Surveillance, Epidemiology, and End Results database; or 0.020 per 100,000 according to the Rare Tumors in Pediatric Age Project.^[6,15]^ This helps to explain why so few studies of pancreatic cancer in adolescents have been reported,^[7,10,12,16]^ and this lack of knowledge means that optimal treatments for such patients is unclear. In this analysis of such patients treated at our institution over a 15-year period, we found that radical resection did not increase risk of serious complications or disturb growth and development. And the radical resection may be associated with lower recurrence than enucleation.

Complete surgical resection is the goal of treatment. For adolescents with pancreatic tumors, resection is the preferred initial treatment.^[6,17,18]^ The ability to resect depends on the local extent of the disease, the location, and the existence or absence of metastatic disease.^[3]^ And the choice of radical resection or enucleation depends on the pathological evidence. For malignant pancreatic tumors, radical resection is needed (pancreatic head lesions require PD and lesions of the body and tail require distal pancreatectomy with or without splenectomy). But for benign or borderline pancreatic tumors, the choice still need further discussion.

SPT is a rare epithelial solid tumor of the pancreas that accounts for 2% to 3% of primary pancreatic malignancies.^[19]^ It affects primarily women in their 30s,^[20–22]^ though 22% of cases have been reported to occur in patients aged 19 years and younger.^[21]^ Five patients in our series were diagnosed with SPT, and the male-to-female ratio was 1:4, similar to that reported by Lee et al (1:6.5)^[23]^ and Zampieri et al (1:4).^[24]^ The mean age of SPT patients in our series was 15 years, consistent with other series.^[25,26]^ Prognosis of patients with SPT depends partly on whether the disease is benign or malignant. Malignant disease usually involves one or more of the following: perineural invasion, angioinvasion, peripancreatic soft tissue invasion, capsular invasion, lymph node involvement, adjacent organ invasion, and distant metastasis.^[27]^ These aggressive characteristics of SPTs are usually associated with worse outcome.^[11,28]^ None of the patients in our series showed invasion outside the pancreas. This may help explain why none of the patients with SPT showed recurrence during follow-up.

PNETs are mainly solitary lesions, 90% of which are benign. Most of these tumors occur in children older than 4 years, although some neonatal cases have been reported.^[2]^ All patients in our series had functioning PNETs and all had hypoglycemia. Insulinomas are the most frequent type of PNET, and have been reported in children as young as 5 years.^[29,30]^ In our series of 5 PNET patients, 3 had insulinomas. By contrast, none of our patients showed preoperative metastasis, even though 10% to 20% of PNET patients may present with metastasis.^[31]^ Two PNET patients in our cohort showed postoperative recurrence, similar to the rate reported in Rojas et al.^[32]^ In our study, one of PNET patients exhibiting multiple endocrine neoplasias. A plenty of measures were needed to detect additional tumors in the pancreas. Preoperative imaging tests (computed tomography/magnetic resonance imaging scan) or endoscopic ultrasound, intraoperative ultrasound and surgeon's own judgments may help to detect and localize the additional pancreatic tumors. Common PNET treatments include both surgery and chemotherapy.^[31]^ Conventional chemotherapeutic agents are usually ineffective against PNETs because they express high levels of somatostatin receptor. However, adolescents have been shown to respond to peptide receptor radionuclide therapy.^[33]^

Two patients in our series (patients 3 and 10) suffered malignant pancreatic tumors, which are rare in adolescents: overall incidence among patients 19 years or younger is 0.18 per million in the United States.^[15]^ Another study analyzed 21 malignant pancreatic tumors over 8 years and reported an estimated annual incidence of 0.20 per million of US children aged 19 years or younger.^[6]^ Surgical resection remains the standard treatment for patients with malignant pancreatic tumors.^[11]^ Both patients in our series underwent classic Whipple radical resection. Portal vein invasion was detected in 1 patient (patient 3), and the portal vein was resected and reconstructed uneventfully, with no recurrence during follow-up. These results are consistent with a report that en bloc vascular resection followed by reconstruction with vein grafts can be effective.^[28]^

The results from our series suggest that in patients with malignant tumors, early complete and even aggressive surgical resection is key to good survival outcomes.^[1–3,15]^ Radical primary intervention is associated with favorable outcomes in patients with no regional or metastatic extension.^[16]^ Indeed, radical resection is associated with better OS and RFS than nonradical alternatives in patients with various types of malignant or benign pancreatic tumors.^[34]^

Nevertheless, several studies suggest that limited surgical resection, such as in the enucleation procedure, may be effective in certain patients. One study reported that 3 female patients with SPT aged 8, 14, and 16 years who had a positive tumor margin were still alive by the end of follow-up, with OS ranging from 19 to 118 months.^[11]^ Several other studies have also suggested that enucleation, rather than radical resection, can provide good long-term survival in SPT patients with positive margins.^[21,32,35]^ Patient selection for enucleation should be based on the results of frozen-section biopsy, macroscopic appearance of the tumor, and location within the pancreas.^[25]^ Our results suggest that enucleation may not be as effective as radical resection, since patients who underwent limited resection in our series experienced a significantly higher rate of relapse and shorter RFS. Interestingly, all 3 patients who experienced recurrence were operated in other institutions and had relapse lesion resected in our institution. This calls for centralization of major pancreatic surgeries which aids in proper decision making and management of these rare but potentially curable tumors. When inexperienced surgeons are diagnosing these rare pancreatic tumors, tertiary referral centers with expertise in pancreatic surgery are urgent needed to improve clinical outcome.

Enucleation has been associated with high risk of postoperative pancreatic leakage in SPT patients.^[25,36]^ In our series, patient 4 experienced a grade B pancreatic fistula after initial enucleation at a local hospital. By contrast, none of the patients in our series who underwent radical resection at our hospital experienced serious complications.

In contrast to treating adults with pancreatic cancer, treating adolescents should aim to minimize effects on growth and development. Therefore, we examined growth and development in our cohort after each type of resection procedure. Analysis of height, weight, and BMI of each patient showed that by the end of follow-up, all patients showed normal growth except for patient 3, who was overweight, and patient 8, who was underweight. The change in patient 3 may partly reflect body weight instability unrelated to the surgical procedure: the patient weighed 75 kg at the end of follow-up, compared with 100 kg before surgery. Exercise and diet control are likely needed to ensure stable, healthy weight in this patient. The underweight of patient 8, in contrast, seems likely to reflect treatment-associated psychological stress (anxiety) and physical stress (wasting disease), exacerbated by the discovery of liver metastasis only 3 months after the initial surgery, probably before the patient's physical status had returned to normal. Taken together, the results with this patient series suggest that radical resection is generally safe in adolescents. Indeed, none of our patients showed surgery-induced diabetes.

Our case series provides insights into treatment outcomes using radical or limited resection in a Chinese cohort of adolescents with pancreatic tumors. Inevitably our results are limited by the small sample size, reflecting the rarity of the disease in adolescents. Nevertheless, we did examine clinical experiences at our hospital over a 15-year period with 11 patients, which is within the range of 10 to 30 patients reported in Western studies. Larger studies with longer follow-up are needed to verify and extend our findings.

In conclusion, our patient series provides evidence from a Chinese medical center that radical resection can be safe and effective for adolescents with pancreatic tumors. The procedure may be associated with lower risk of recurrence and a slightly longer RFS than limited resection (enucleation).
